# Solly on the Human Brain

**Published:** 1848-05

**Authors:** 


SOLLY ON THE HUMAN BRAIN.

We are indebted to those enterprising publishers, Messrs. Lea and Blanchard, for a beautiful copy of this elaborate work. The author has given a thorough view of this important organ, its anatomy, physiology, pathology and diseases, and we are glad that his treatise has been rendered available to American readers by this publication. Any student who is ambitious of an intimate acquaintance with the brain must consult the work of Mr. Solly, which is presented, in this edition, in a form worthy of its high character.

